# First dose COVID-19 vaccine coverage amongst adolescents and children in England: an analysis of 3.21 million patients' primary care records in situ using OpenSAFELY

**DOI:** 10.12688/wellcomeopenres.18735.1

**Published:** 2023-02-10

**Authors:** Lisa E. Hopcroft, Helen J. Curtis, Andrew D. Brown, William J. Hulme, Colm D. Andrews, Caroline E. Morton, Peter Inglesby, Jessica Morley, Amir Mehrkar, Sebastian C. Bacon, Rosalind M. Eggo, Viyaasan Mahalingasivam, Edward P. K. Parker, Laurie A. Tomlinson, Christopher Bates, Jonathan Cockburn, John Parry, Frank Hester, Sam Harper, Ben Goldacre, Alex J. Walker, Brian MacKenna

**Affiliations:** 1 Bennett Institute for Applied Data Science, Nuffield Department of Primary Care Health Sciences, University of Oxford, Oxford, OX2 6GG, UK; 2 London School of Hygiene & Tropical Medicine, London, WC1E 7HT, UK; 3 TPP, TPP House, 129 Low Lane, ,Horsforth, Leeds, LS18 5PX, UK

**Keywords:** Covid-19, Primary Health Care, Public Health, vaccine

## Abstract

**Background:** The coronavirus disease 2019 (COVID-19) vaccination programme in England was extended to include all adolescents and children by April 2022. The aim of this paper is to describe trends and variation in vaccine coverage in different clinical and demographic groups amongst adolescents and children in England.

**Methods:** With the approval of NHS England, a cohort study was conducted of 3.21 million children and adolescents’ records in general practice in England, *in situ* and within the infrastructure of the electronic health record software vendor TPP using OpenSAFELY. Vaccine coverage across various demographic (sex, deprivation index and ethnicity) and clinical (risk status) populations is described.

**Results:** Coverage is higher amongst adolescents than it is amongst children, with 53.5% adolescents and 10.8% children having received their first dose of the COVID-19 vaccine. Within those groups, coverage varies by ethnicity, deprivation index and risk status; there is no evidence of variation by sex.

**Conclusion: **First dose COVID-19 vaccine coverage is shown to vary amongst various demographic and clinical groups of children and adolescents.

## Introduction

By April 2022, the coronavirus disease 2019 (COVID-19) vaccination programme in England had been expanded to include all adolescents (12–15 year olds) and children (5–11 year olds). Invitations were extended to those considered clinically vulnerable or living with a vulnerable adult first (adolescents in August 2021 and children in January 2022) with all others being invited soon after (adolescents in September 2021 and children in April 2022). We have extended our existing COVID-19 vaccine analysis pipeline ^1^, implemented using the OpenSAFELY platform, to include adolescents and children ^2^. Our analysis queries the primary care data of 3.21m adolescents and children, a (~40%) subset of the entire population of adolescents and children in England (specifically, this subset is all 5–15 year olds who belong to a GP practice using TPP SystmOne EHR software).

## Methods

### Study design

A retrospective cohort study was conducted using general practice (GP) primary care EHR data from all England GP practices supplied by the EHR vendor TPP. The cohort study began on 7 ^th^ December 2020 (to capture the start of the national vaccination campaign which began on 8 ^th^ December 2020) and ended on 10 August 2022.

### Data access and verification

Access to the underlying identifiable and potentially re-identifiable pseudonymised electronic health record data is tightly governed by various legislative and regulatory frameworks, and restricted by best practice. The data in OpenSAFELY is drawn from General Practice data across England where TPP is the Data Processor. TPP developers (CB, JC, JP, FH, and SH) initiate an automated process to create pseudonymised records in the core OpenSAFELY database, which are copies of key structured data tables in the identifiable records. These are linked onto key external data resources that have also been pseudonymised via SHA-512 one-way hashing of NHS numbers using a shared salt. Bennett Institute for Applied Data Science developers and PIs (CEM, SCB, AJW, WJH, HJC, PI) holding contracts with NHS England have access to the OpenSAFELY pseudonymised data tables as needed to develop the OpenSAFELY tools. These tools in turn enable researchers with OpenSAFELY Data Access Agreements to write and execute code for data management and data analysis without direct access to the underlying raw pseudonymised patient data, and to review the outputs of this code.

### Study population

All patients alive and registered with a general practice using TPP in England on 10 August 2022 and aged between 5 and 15 on that same date were included in this study. Patients without a recorded sex were excluded.

### COVID-19 vaccine status

Vaccination information is transmitted back to patients’ primary care records in the days following vaccine administration in a designated centre. Which patients had any recorded COVID-19 vaccine administration code in their primary care record (Pfizer-BioNTech mRNA vaccine, AstraZeneca-Oxford vaccine or Moderna vaccine) was ascertained. The latest available date of vaccinations recorded in the most recent comparable OpenSAFELY–TPP database build were included for those vaccinated up to 10 August 2022. All counts are rounded to the nearest 7.

### Key demographic and clinical characteristics of vaccinated groups

Patient demographics defined by the national reporting specification (for example, ethnicity) were extracted. Demographics not defined by the specification, including the level of deprivation, were also extracted. Deprivation was measured by the Index of Multiple Deprivation (IMD, in quintiles, with higher values indicating greater deprivation), derived from the patient’s postcode at Lower Super Output Area. Patients with missing data were grouped into an unknown category.

### Risk status

The patients who are 'In a risk group' have been identified using the criteria in Table 4 of The Green Book and codelists from SARS-CoV-2 (COVID-19) Vaccine Uptake Reporting Specification Collection 2020/2021 (v1.5.3) as distributed by PRIMIS. This includes patients with: immunosuppression; chronic kidney disease; chronic liver disease; chronic heart disease; chronic respiratory disease; chronic neurological disease (including stroke/TIA, cerebral palsy, or MS); asplenia or dysfunction of the spleen; asthma; diabetes; severe mental illness; learning disabilities and pregnancy.

### Software availability

All code for the full data management pipeline—from raw data to completed results for this analysis—and for the OpenSAFELY platform as a whole is available for review at GitHub and archived in Zenodo ^3^.

Data management and analysis was performed using the OpenSAFELY software libraries and Python, both implemented using Python 3, with additional analyses carried out using R. Code for data management and analysis as well as codelists is archived online ( https://github.com/opensafely/nhs-covid-vaccination-coverage/tree/1.46.1).

## Results

**Figure 1.**
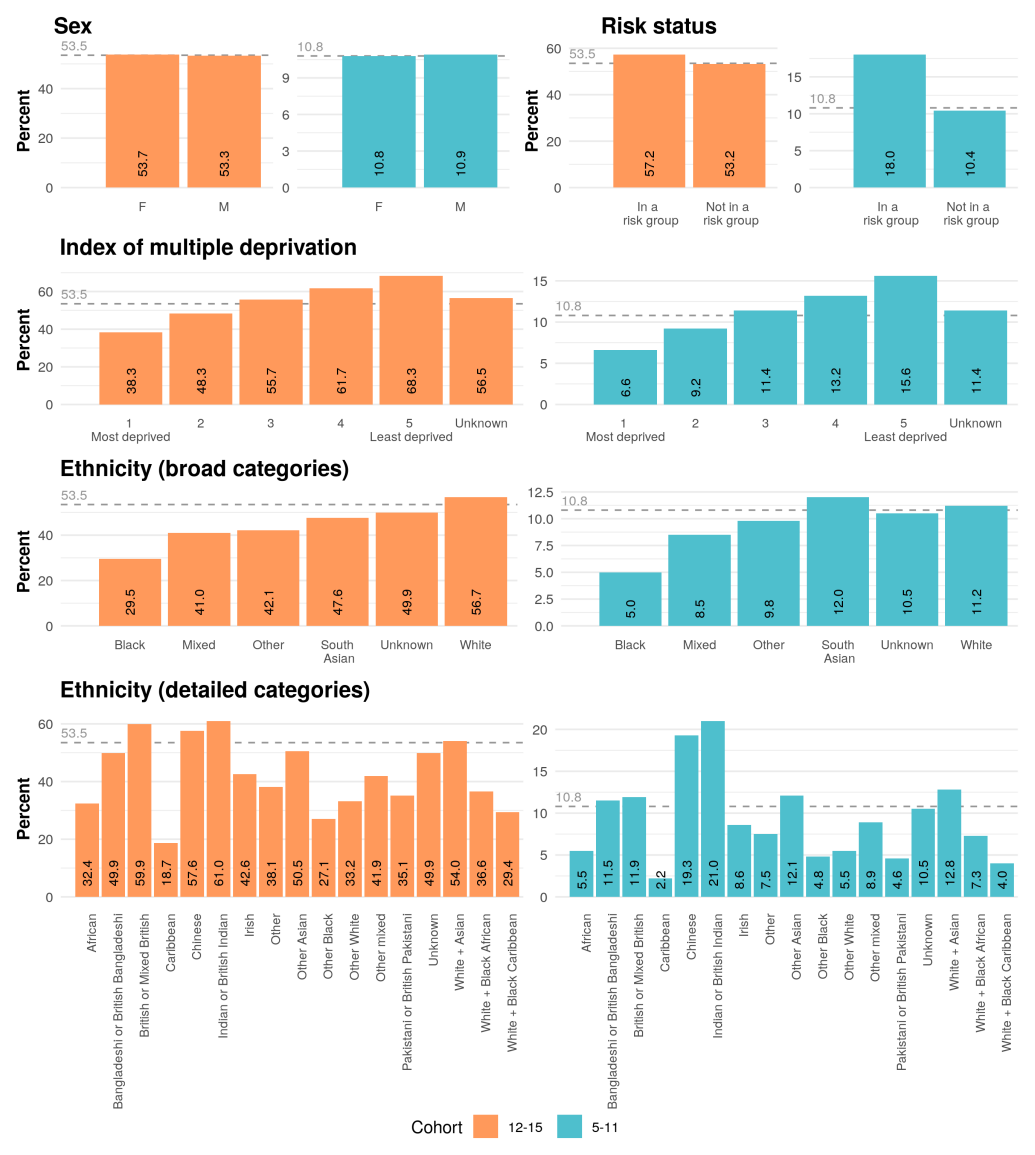
The coverage of first dose COVID-19 vaccination amongst adolescents (12–15) and children (5–11) in England, broken down by demographic (sex, index of multiple deprivation and ethnicity) and clinical characteristics (risk status), as of 10 August 2022. Coverage is calculated as a percentage of the whole cohort. The overall coverage for each cohort is indicated with a dashed grey line on each plot. Vaccine campaign length: 52 weeks for vulnerable adolescents (began 6 August 2021); 46 weeks for all other adolescents (began 21 September 2021). Vaccine campaign length: 27 weeks for vulnerable children (began 30 January 2022); 18 weeks for all other children (began 4 April 2022).

Figure 1 summarises first dose COVID-19 vaccine coverage for relevant demographic and clinical populations, as calculated by our most recent analysis. As of 10th August 2022, 53.5% (633,122 of 1,183,931) adolescents and 10.8% (219,163 of 2,022,167) children had received their first COVID-19 vaccination dose. No variation by sex was observed.

Disparities amongst ethnic groups observed previously in adults are also observed in the under 16 cohorts ^1^. 56.7% (492,905 of 868,980) of White adolescents have received a first dose compared to 29.5% (12,159 of 41,258) of Black adolescents. 12.0% (23,506 of 195,132) of South Asian children have received a first dose compared to 5.0% (3,269 of 65,716) of Black children. Coverage is particularly low in the Caribbean population: in this group, 18.7% (875 of 4,690) adolescents and 2.2% (161 of 7,175) children have been vaccinated. There is noticeably higher than average uptake in other ethnic groups: 61.0% (21,469 of 35,168) Indian or British Indian adolescents and 21.0% (13,566 of 64,589) Indian or British Indian children have received their first dose. Vaccine coverage is similarly high amongst the Chinese population: 57.6% (3,934 of 6,832) and 19.3% (2,114 of 10,934) amongst Chinese adolescents and children respectively.

Variation in vaccine coverage by deprivation previously observed amongst adults persists in the under 16 cohorts. First dose coverage is lower in areas of high deprivation: 38.3% (106,743 of 278,796) adolescents living in the most deprived quintile have received their first dose compared to 68.3% (141,540 of 207,158) adolescents living in the least deprived quintile. A similar pattern was observed in the 5–11 year old population, with 15.6% (51,443 of 329,077) and 6.6% (32,207 of 484,596) uptake in the least and most deprived quintile respectively.

There is some evidence that coverage is higher amongst children identified as at higher risk of severe COVID-19 (and therefore invited for their first vaccination at an earlier date). First dose uptake is 57.2% (51,373 of 89,859) amongst adolescents “in a risk group”, compared to 53.2% (581,749 of 1,094,072) amongst those “not in a risk group”; similarly, 18.0% (21,350 of 118,839) of children “in a risk group” have received their first dose, compared to 10.4% (197,813 of 1,903,328) of children “not in a risk group”.

## Discussion

Overall, first dose COVID-19 vaccine coverage is lower amongst 5–15 year olds than it is amongst the adult (over 16) population. Coverage amongst children is particularly low: a higher percentage of adolescents have received their first dose in all demographic and clinical subgroups. In both age groups, coverage is shown to vary amongst all but one of the demographic and clinical groups examined (sex being the only breakdown that does not exhibit a difference). Demographic disparities previously observed amongst the adult population are also observed for 5–15 year olds. There is some evidence that those identified as being “in a risk group” are more likely to be vaccinated; this is more apparent in the 5–11 age group than the 12–15 age group.

For context, cumulative coverage figures demonstrate that coverage is continuing to increase over time, particularly amongst children. We encourage readers to view the full report to inform vaccination campaigns locally and address any inequalities in vaccination coverage.

### Information governance and ethical approval

NHS England is the data controller for OpenSAFELY-TPP; TPP is the data; all study authors using OpenSAFELY have the approval of NHS England. This implementation of OpenSAFELY is hosted within the TPP environment which is accredited to the ISO 27001 information security standard and is NHS IG Toolkit compliant NHS IG Toolkit compliant.

Patient data has been pseudonymised for analysis and linkage using industry standard cryptographic hashing techniques; all pseudonymised datasets transmitted for linkage onto OpenSAFELY are encrypted; access to the platform is via a virtual private network (VPN) connection, restricted to a small group of researchers; the researchers hold contracts with NHS England and only access the platform to initiate database queries and statistical models; all database activity is logged; only aggregate statistical outputs leave the platform environment following best practice for anonymisation of results such as statistical disclosure control for low cell counts.

The OpenSAFELY research platform adheres to the obligations of the UK General Data Protection Regulation (GDPR) and the Data Protection Act 2018. In March 2020, the Secretary of State for Health and Social Care used powers under the UK Health Service (Control of Patient Information) Regulations 2002 (COPI) to require organisations to process confidential patient information for the purposes of protecting public health, providing healthcare services to the public and monitoring and managing the COVID-19 outbreak and incidents of exposure; this sets aside the requirement for patient consent. This was extended in November 2022 for the NHS England OpenSAFELY COVID-19 research platform. In some cases of data sharing, the common law duty of confidence is met using, for example, patient consent or support from the Health Research Authority Confidentiality Advisory Group.

Taken together, these provide the legal bases to link patient datasets on the OpenSAFELY platform. GP practices, from which the primary care data are obtained, are required to share relevant health information to support the public health response to the pandemic, and have been informed of the OpenSAFELY analytics platform.

This study was approved by the Health Research Authority (REC reference 20/LO/0651) and by the LSHTM Ethics Board (reference 21863).

## Data Availability

All data were linked, stored and analysed securely within the OpenSAFELY platform https://opensafely.org/. Data include pseudonymized data such as coded diagnoses, medications and physiological parameters. No free text data are included. All code is shared openly for review and re-use under MIT open license ( https://github.com/opensafely/nhs-covid-vaccination-coverage/tree/1.46.1). Detailed pseudonymised patient data is potentially re-identifiable and therefore not shared. We rapidly delivered the OpenSAFELY data analysis platform without prior funding to deliver timely analyses on urgent research questions in the context of the global Covid-19 health emergency: now that the platform is established we are developing a formal process for external users to request access in collaboration with NHS England; details of this process are available at OpenSAFELY.org/onboarding-new-users.

## Software availability

- **Source code available from:** https://github.com/opensafely/nhs-covid-vaccination-coverage/tree/1.46.1
- **Archived source code at time of publication:** https://doi.org/10.5281/zenodo.7547923 ^3^.
- **License:** MIT
