# Supplementary material for: First dose COVID-19 vaccine coverage amongst adolescents and children in England: an analysis of 3.21 million patients' primary care records in situ using OpenSAFELY
Source: Wellcome Open Res. 2023 Jun 9;8:70. Originally published 2023 Feb 10. [Version 2] doi: 10.12688/wellcomeopenres.18735.2 (PMC10280033; doi:10.12688/wellcomeopenres.18735.2)
Supplement: Peer review history

# Peer review: Reviewer response for version 2

**DOI:** 10.21956/wellcomeopenres.21646.r59783

**Published:** 2023-06-12

**Authors:** Kimberley Foley

**Affiliations:** 1 Department of Primary Care and Public Health, Imperial College London, London, England, UK

Many thanks to the authors for the detailed response to my review and for addressing all of my comments and suggestions. I have no further comments.

Is the work clearly and accurately presented and does it cite the current literature?

Partly

If applicable, is the statistical analysis and its interpretation appropriate?

No

Are all the source data underlying the results available to ensure full reproducibility?

Yes

Is the study design appropriate and is the work technically sound?

Partly

Are the conclusions drawn adequately supported by the results?

Partly

Are sufficient details of methods and analysis provided to allow replication by others?

Partly

Reviewer Expertise:

Epidemiology, health services research

I confirm that I have read this submission and believe that I have an appropriate level of expertise to confirm that it is of an acceptable scientific standard.

# Peer review: Reviewer response for version 2

**DOI:** 10.21956/wellcomeopenres.21646.r59784

**Published:** 2023-06-12

**Authors:** Ruth H. Jack

**Affiliations:** 1 Centre for Academic Primary Care, Lifespan and Population Health, School of Medicine, University of Nottingham, Nottingham, UK

The authors have addressed all raised issues, I have no further comments.

Is the work clearly and accurately presented and does it cite the current literature?

Yes

If applicable, is the statistical analysis and its interpretation appropriate?

Yes

Are all the source data underlying the results available to ensure full reproducibility?

Partly

Is the study design appropriate and is the work technically sound?

Yes

Are the conclusions drawn adequately supported by the results?

Yes

Are sufficient details of methods and analysis provided to allow replication by others?

Yes

Reviewer Expertise:

Epidemiology using large databases of electronic health records

I confirm that I have read this submission and believe that I have an appropriate level of expertise to confirm that it is of an acceptable scientific standard.

# Peer review: Reviewer response for version 1

**DOI:** 10.21956/wellcomeopenres.20774.r57564

**Published:** 2023-05-30

**Authors:** Ruth H. Jack

**Affiliations:** 1 Centre for Academic Primary Care, Lifespan and Population Health, School of Medicine, University of Nottingham, Nottingham, UK

This research note describes the (early) uptake of the COVID-19 vaccination in children (aged 5 to 11 years) and adolescents (aged 12 to 15 years) in England, using routinely collected primary care electronic health records from OpenSAFELY.  A large cohort of over 3 million people from August 2022 were included and vaccination status examined in the different age groups and by other characteristics. Uptake varied by age, risk status, deprivation and ethnicity, but not sex.

 This is a useful piece of work describing how the COVID-19 vaccine uptake varied in children and adolescents, enabling comparisons with similar data for adults, and determining which groups should be focused on to increase vaccination rates.

 The Discussion needs to highlight more that the uptake in the different age and risk groups is likely to be affected by the vaccine campaign length, rather than this being a footnote in the figure, and referenced in the external link. It will be interesting to see whether these variations persist in children when the vaccine had been available for a longer period, or whether the differences narrow.

 I have a few more specific comments below.

*Abstract - Results*. Consider adding “by August 2022” at the end of the first sentence.

*Study design*.  Instead of defining the cohort study beginning and ending, it might be clearer to describe “the vaccine data were available between 7 ^th^ December…”.

*Key demographic and clinical characteristics of vaccinated groups*. The description of the IMD quintiles implies that a higher quintile score relates to greater deprivation, while the figures show that quintile 5 is the least deprived quintile.

*Figure 1*. Consider labelling the key with ‘age’ or ‘years’ for clarity.

 Is the ‘British or Mixed British’ group from the broad White group? It would be helpful to detail which of the broad groups the detailed categories belong to somewhere in the text or figure.

*Information governance and ethical approval*. “NHS IG Toolkit compliant” is duplicated.

Is the work clearly and accurately presented and does it cite the current literature?

Yes

If applicable, is the statistical analysis and its interpretation appropriate?

Yes

Are all the source data underlying the results available to ensure full reproducibility?

Partly

Is the study design appropriate and is the work technically sound?

Yes

Are the conclusions drawn adequately supported by the results?

Yes

Are sufficient details of methods and analysis provided to allow replication by others?

Yes

Reviewer Expertise:

Epidemiology using large databases of electronic health records

I confirm that I have read this submission and believe that I have an appropriate level of expertise to confirm that it is of an acceptable scientific standard.

# Response

**Published:** 2023-06-06

**Authors:** Helen Curtis

**Affiliations:** University of Oxford, UK

-- Thank you for taking the time to review our work and for the positive comments. In version 2 of the paper we have addressed your comments as follows:

 The Discussion needs to highlight more that the uptake in the different age and risk groups is likely to be affected by the vaccine campaign length, rather than this being a footnote in the figure, and referenced in the external link. It will be interesting to see whether these variations persist in children when the vaccine had been available for a longer period, or whether the differences narrow.

 -- We have expanded on this in the Discussion as suggested.  

 I have a few more specific comments below.  

 Abstract - Results.

 Consider adding “by August 2022” at the end of the first sentence.

 -- We have added this as suggested.  

 Study design.  Instead of defining the cohort study beginning and ending, it might be clearer to describe “the vaccine data were available between 7th December…”.

 -- We have rephrased this.  

 Key demographic and clinical characteristics of vaccinated groups. The description of the IMD quintiles implies that a higher quintile score relates to greater deprivation, while the figures show that quintile 5 is the least deprived quintile.

 -- We have removed the confusing mention of this from the methods.

 Figure 1. Consider labelling the key with ‘age’ or ‘years’ for clarity.

 -- We have added "years" to the figure legend.  

 Is the ‘British or Mixed British’ group from the broad White group? It would be helpful to detail which of the broad groups the detailed categories belong to somewhere in the text or figure.

 -- We have now described this in the figure legend.  

 Information governance and ethical approval. “NHS IG Toolkit compliant” is duplicated.

 -- Thank you, we have removed the repeated phrase.

# Peer review: Reviewer response for version 1

**DOI:** 10.21956/wellcomeopenres.20774.r54905

**Published:** 2023-03-07

**Authors:** Kimberley Foley

**Affiliations:** 1 Department of Primary Care and Public Health, Imperial College London, London, England, UK

This work presents interesting findings about the COVID-19 vaccine coverage for children and adolescents. This is a large study using population-based data, but there are several aspects of this work that could be clarified, which I will highlight below:

 The introduction to this research note is very brief, but additional context would be useful. For example, information on the status of government restrictions/social distancing measures in relation to the timing of offering vaccines to children and adolescents might be helpful. The aim of the paper was included in the "background" section of the abstract, but was not repeated in the introduction and should also be included here.

 The methods were briefly outlined, but again, more detail would be helpful. I appreciate this is a research note and therefore will be less detailed than a full research article, but it appears to currently be well within the word limits so key areas could be expanded. For example, it appears the initial vaccine cohort using the OpenSAFELY platform included individuals belonging to GP practices using both TPP and EMIS, but this subset is only TPP - are there likely to be any expected differences in the study population that may affect generalisability?

 I would appreciate it if the authors could further explain/justify why the study population included everyone alive/registered with a GP practice at the end date of the study period.

 In the methods section it would be helpful to see a full list of the key demographic and clinical characteristics included in this study. It would also be useful to return to this list in the discussion to discuss any that were not included that may be associated with vaccination of child/adolescents (e.g. parent/carer vaccination status?). In reading the methods, I wasn't entirely clear as to whether data on household risk status was available. Were the authors able to identify children/adolescents living with a vulnerable adult, and therefore were these children/adolescents included in the "in a risk group" category? It would be helpful to clarify this point and if these children/adolescents were not included in the "in a risk group", the implications of this could be discussed further in the discussion section.

 The results present descriptive data in a series of figures. However as mentioned in the footnote to the figure, vaccine eligibility for each group (at risk vs not, children vs adolescents) varied over time. I wondered whether the relationship between these groups and demographic factors varied? (e.g. vaccine uptake for children aged 5-11 and "not at risk" was lower, but this may be due to less time eligible - would the differences observed in ethnicity/IMD based on vaccination status be greater for this group? Or to ask a separate question, does the proportion of children/adolescents "at risk" vary among different ethnic groups?) I would appreciate justification in the methods why these groups were not analysed separately. Additionally, as proportions are being compared in this research note, it would be useful to include the relevant statistics to support the statements made in the text. 

 In the discussion, some of the main findings of this work are clearly summarised, but what are the implications of this work? The authors helpfully point out that vaccine coverage is increasing over time, but readers are encouraged to view the full report. Which details of the full report would be useful for readers to understand? What are the conclusions/lessons to be learned from this research?

 Many thanks for the opportunity to review this interesting report and I hope these comments are helpful to the authors.

Is the work clearly and accurately presented and does it cite the current literature?

Partly

If applicable, is the statistical analysis and its interpretation appropriate?

No

Are all the source data underlying the results available to ensure full reproducibility?

Yes

Is the study design appropriate and is the work technically sound?

Partly

Are the conclusions drawn adequately supported by the results?

Partly

Are sufficient details of methods and analysis provided to allow replication by others?

Partly

Reviewer Expertise:

Epidemiology, health services research

I confirm that I have read this submission and believe that I have an appropriate level of expertise to confirm that it is of an acceptable scientific standard, however I have significant reservations, as outlined above.

# Response

**Published:** 2023-06-09

**Authors:** Helen Curtis

**Affiliations:** University of Oxford, UK

Thank you for taking the time to review our work and for the largely positive review and constructive suggestions. We have now submitted a revised version of the article and please see responses to specific points below.

 "The introduction to this research note is very brief, but additional context would be useful. For example, information on the status of government restrictions/social distancing measures in relation to the timing of offering vaccines to children and adolescents might be helpful. The aim of the paper was included in the "background" section of the abstract, but was not repeated in the introduction and should also be included here." -- We have added the aim to the Introduction but we think that otherwise sufficient detail has been supplied for a research note.

 "The methods were briefly outlined, but again, more detail would be helpful. I appreciate this is a research note and therefore will be less detailed than a full research article, but it appears to currently be well within the word limits so key areas could be expanded. For example, it appears the initial vaccine cohort using the OpenSAFELY platform included individuals belonging to GP practices using both TPP and EMIS, but this subset is only TPP - are there likely to be any expected differences in the study population that may affect generalisability?" -- We have added a note on TPP coverage to the Discussion and in the Methods we have expanded on the inclusion criteria and demographic groups section as suggested below. All code is openly shared for inspection and re-use by any interested reader in more detailed information on the methods.

 "I would appreciate it if the authors could further explain/justify why the study population included everyone alive/registered with a GP practice at the end date of the study period." -- We have expanded on this as suggested.

 "In the methods section it would be helpful to see a full list of the key demographic and clinical characteristics included in this study. It would also be useful to return to this list in the discussion to discuss any that were not included that may be associated with vaccination of child/adolescents (e.g. parent/carer vaccination status?). In reading the methods, I wasn't entirely clear as to whether data on household risk status was available. Were the authors able to identify children/adolescents living with a vulnerable adult, and therefore were these children/adolescents included in the "in a risk group" category? It would be helpful to clarify this point and if these children/adolescents were not included in the "in a risk group", the implications of this could be discussed further in the discussion section." -- We have expanded on this in the Methods and Discussion as suggested.

 "The results present descriptive data in a series of figures. However as mentioned in the footnote to the figure, vaccine eligibility for each group (at risk vs not, children vs adolescents) varied over time. I wondered whether the relationship between these groups and demographic factors varied? (e.g. vaccine uptake for children aged 5-11 and "not at risk" was lower, but this may be due to less time eligible - would the differences observed in ethnicity/IMD based on vaccination status be greater for this group? Or to ask a separate question, does the proportion of children/adolescents "at risk" vary among different ethnic groups?) I would appreciate justification in the methods why these groups were not analysed separately. " -- We have now expanded on this in the Discussion.

 "Additionally, as proportions are being compared in this research note, it would be useful to include the relevant statistics to support the statements made in the text. " -- We have not implemented statistical tests in this short research note as the results presented are sufficient to meet the aims of exploring trends and variation, and are consistent with our previous papers on adult vaccine coverage and with the data presented in our linked full report.

 "In the discussion, some of the main findings of this work are clearly summarised, but what are the implications of this work? The authors helpfully point out that vaccine coverage is increasing over time, but readers are encouraged to view the full report. Which details of the full report would be useful for readers to understand? What are the conclusions/lessons to be learned from this research?" -- We have expanded this part of the Discussion as suggested.
